# Supplementary material for: Intrauterine exposure to perfluorinated compounds and overweight at age 4: A case-control study
Source: PLoS One. 2020 Mar 16;15(3):e0230137. doi: 10.1371/journal.pone.0230137 (PMC7075550; doi:10.1371/journal.pone.0230137)
Supplement: Attachment — Submitted filename: Response to reviewers.docx [file pone.0230137.s001.docx]

# Response to academic editor and reviewers

Please find our response to each point raised below. The indicated page numbers and lines refer to the marked-up version of the revised manuscript.

## Journal Requirements

*1) In ethics statement in the manuscript and in the online submission form, please provide additional information about the patient records used in your retrospective study, in particular regarding children data. Specifically, please ensure that you have discussed whether all data were fully anonymized before you accessed them and/or whether the IRB or ethics committee waived the requirement for informed consent. If patients provided informed written consent to have data from their medical records used in research, please include this information.*

AU: No patient records were used. The children’s data comes from a research questionnaire that their parents agreed to fill out at the regular 4-year health examination, combined with weight and height measured at the same time by the nurse (clarified at p. 4; line 82).

The ethics committee waived the requirement for informed consent and all analyses were performed on pseudonymized data. This has now been added to the text (p. 5; line 101).

*2. In your Methods section, please provide additional information about the participant inclusion method and the demographic details of your participants. Please ensure you have provided sufficient details to replicate the analyses such as: a) a description of any inclusion/exclusion criteria that were applied to participant inclusion, b) a table of relevant demographic details, c) a statement as to whether your sample can be considered representative of a larger population.*

AU: Participants were recruited by their pediatric nurse in connection with the routine 4-years health exam. This has now been added to the text (p. 4; line 82). The demographic details of the study sample are presented in Table 1. We have clarified this in the table heading.

 a) Inclusion and exclusion criteria are stated under Study population (p. 4-5).

 b) The demographics that was available is presented in Table 1. The table heading has been
 clarified.

c) The sample can be considered representative for children who are born full term of
 parents born in Sweden. In this context, it should be mentioned that the proportion of
 women who smoke during pregnancy has declined since the data in the study was
 collected. This has been added under Strengths and limitations (p. 15; line 317).

*3. We note that you have indicated that data from this study are available upon request. PLOS only allows data to be available upon request if there are legal or ethical restrictions on sharing data publicly.*
AU: Data contains sensitive personal information and cannot be shared publicly for legal and ethical reasons.

Reviewers' comments

**Reviewer #1:**

Major comments:

*1. Please provide more details (formulations) about calculating the OR between quartiles of maternal serum levels and child overweight at age 4. It is not clear to me how you model the logistic regression.*

AU: In the logistic regressions, where the outcome was overweight at 4 years of age (yes/no), we took two analytical approaches:

i) On the full dataset we included the following variables

- Risk strata
- Difference from strata–specific mean
- Sex

ii) Within each risk strata (i.e. stratified analysis), we included the following variables

- Difference from strata–specific mean
- Sex

In both settings, we included a random term for mother to account for the hierarchical nature of the data.

We have carefully reconsidered each formulation in the text and make the judgement that all the information is there. Although we do respect Reviewer 1’s viewpoint, we would like to refer to Reviewer 2 stating that “All statistical analyses and analytical measurements conducted in the research were described in detail in the manuscript in a sufficient manner to allow for replication by other researchers”.

Please note that when reading the Methods section, we found that a sentence has dropped out of the text. Thus, we have added: “We explored interactions between exposures and risk strata in the full-dataset analyses but the terms were non-significant and therefore not included in the final models.” (p. 8; line 176).

*2. From Table 1, we can see there are obvious differences between the case group and control group in terms of the levels of the risk factors. Could you demonstrate that the differences are negligible or won't affect the final result?*

AU: Overweight is a multifactorial disease that is affected by several risk factors. Thus, it was expected that the cases would have more present risk factors than the controls. Potential confounding caused by the level of these risk factors was handled by adjusting for the difference in risk score from the strata-specific mean in the final models. However, as in all observational studies, bias due to residual confounding cannot be ruled out.

Minor comments:

*1. Please give the full name of "OR" when it first appeared.*

AU: A mistake on our behalf - thanks for pointing it out. Odds ratio has been spelled out both in the abstract and in the main text. We did not use it that many times and hence chose to spell it out rather than abbreviate it.

*2. Lines 127 and 128: What do you mean by “All cases (n=416) were included and matched with 2 controls per case (n=832) from the same risk strata, with further matching on sex."? Where were the numbers 416 and 832 derived from and what were they used for?*

AU: The 416 children were the overweight children with complete data on covariates in the risk model, and the 832 their matched controls. These were retained for the final analyses. We have combined this paragraph with that above it and added “retained for the final analyses” to clarify (p. 6; line 131).
 *3. Table 3 and Table 4: Labelling sample size as "Cases" is confusing as it include both "cases" and "controls". "Samples" would be better.*

AU: Well spotted! We have changed cases to simply “n” in both tables.

**Reviewer #2:**

*I am interested to know if you plan to follow-up with this same study population as the children age to record/monitor their BMI status through puberty and into young adulthood.*

AU: No, we are not following this specific population. However, we are planning similar studies in Ronneby municipality in southern Sweden where the population has been heavily exposed to contaminated drinking water after decades of leakage of firefighting foams from an airport into the ground water. We are currently establishing a mother-child cohort of 300 dyads where we collect maternal serum from early pregnancy and onwards, as well as breastmilk and serum from the child. We consider this a more relevant setting because of the small exposure contrasts in the general population. In parallel, register-based studies utilizing growth charts from the child health care in Ronneby are ongoing. The first results are expected towards the end of the year.
